# Automatic artefact removal in a self-paced hybrid brain- computer interface system

**DOI:** 10.1186/1743-0003-9-50

**Published:** 2012-07-27

**Authors:** Xinyi Yong, Mehrdad Fatourechi, Rabab K Ward, Gary E Birch

**Affiliations:** 1 Department of Electrical and Computer Engineering, University of British Columbia, 2356 Main Mall, Vancouver, V6T1Z4 Canada; 2 Neil Squire Society, 220 - 2250 Boundary Road, Burnaby, V5M3Z3 Canada

## Abstract

**Background:**

A novel artefact removal algorithm is proposed for a self-paced hybrid brain-computer interface (BCI) system. This hybrid system combines a self-paced BCI with an eye-tracker to operate a virtual keyboard. To select a letter, the user must gaze at the target for at least a specific period of time (dwell time) and then activate the BCI by performing a mental task. Unfortunately, electroencephalogram (EEG) signals are often contaminated with artefacts. Artefacts change the quality of EEG signals and subsequently degrade the BCI’s performance.

**Methods:**

To remove artefacts in EEG signals, the proposed algorithm uses the stationary wavelet transform combined with a new adaptive thresholding mechanism. To evaluate the performance of the proposed algorithm and other artefact handling/removal methods, semi-simulated EEG signals (i.e., real EEG signals mixed with simulated artefacts) and real EEG signals obtained from seven participants are used. For real EEG signals, the hybrid BCI system’s performance is evaluated in an online-like manner, i.e., using the continuous data from the last session as in a real-time environment.

**Results:**

With semi-simulated EEG signals, we show that the proposed algorithm achieves lower signal distortion in both time and frequency domains. With real EEG signals, we demonstrate that for dwell time of 0.0s, the number of false-positives/minute is 2 and the true positive rate (TPR) achieved by the proposed algorithm is 44.7%, which is more than 15.0% higher compared to other state-of-the-art artefact handling methods. As dwell time increases to 1.0s, the TPR increases to 73.1%.

**Conclusions:**

The proposed artefact removal algorithm greatly improves the BCI’s performance. It also has the following advantages: a) it does not require additional electrooculogram/electromyogram channels, long data segments or a large number of EEG channels, b) it allows real-time processing, and c) it reduces signal distortion.

## Background

A brain-computer interface (BCI) system allows humans to use their brain signals (such as EEG) to control various devices such as a virtual keyboard [1-3], a functional electrical stimulator [4], an orthosis [5], amongst others. BCIs can be operated in a synchronized mode or an asynchronous (self-paced) mode [6]. In a synchronized BCI system, the periods when a user can control the system are determined by the system itself. The system usually sends an external cue to the user and the user must then issue a control command within a window of opportunity provided by the system. This limits the use of a synchronized BCI system in practical applications. A self-paced BCI system, on the other hand, allows users to control the system whenever they desire. Hence, the users have a more natural and flexible means for controlling an object [6].

Designing a self-paced BCI system with high performance is associated with two major challenges. They are: 

1. identifying the user’s intentional control (IC) state reliably [IC periods are periods when the user intends to issue control] and

2. reducing the number of false activations (false positives during the no control (NC) periods). [NC periods are periods when the user does not intend to activate the system such as when he/she is obtaining information from the computer screen, thinking about a problem, talking, resting, etc].

NC periods are usually much longer compared to IC periods. As a high number of false positives can result in user frustration, it is especially important to design a system that generates a very low (ideally zero) number of false positives.

It is not easy and straightforward to apply existing pure (i.e., non-hybrid) self-paced BCI systems to operate a practical system such as a virtual keyboard. The reason is that these systems can only recognize a limited number of mental tasks as unique IC commands (mostly one or two). This number is much smaller than the number of letters used in spelling applications. Furthermore, most self-paced BCI systems generate a large number of false positives per minute on average, which is not suitable for most practical applications.

To overcome the above problems, in [7] we have proposed a hybrid system that combines a self-paced BCI with an eye-tracker to operate a virtual keyboard. Our proposed hybrid BCI system also successfully overcomes the ‘Midas Touch’ problem, which is a major problem experienced by conventional eye-gaze interfaces, and results in a significantly smaller false positives generated per minute [7]. The ‘Midas Touch’ problem is the difficulty of determining whether or not the user is intending to select a certain object as the user might be gazing at the object for reasons other than to enter it [8].

As the hybrid BCI system relies on eye movements to control the cursor, it is no surprise that the EEG signals in the system are more contaminated with ocular artefacts compared to EEG signals in a pure BCI system. Also as in other BCI systems, EEG signals are also contaminated with artefacts caused by muscle activities, power line interference, and electrode movements [9]. These artefacts can affect the performance of the system in several ways. In particular, they can: 

1. significantly reduce the amount of data available for designing the system;

2. result in false positives during the NC periods and

3. decrease the true positive rate of the system.

Although some studies have clearly shown that artefacts affect the performance of pure self-paced BCI systems [10,11], little attention has been paid to handle artefacts so far.

In this paper, to minimize the effects of artefacts and improve the performance of our hybrid BCI, we propose a new artefact removal algorithm. The proposed artefact removal algorithm is integrated with our artefact detection algorithm proposed in [12]. Both algorithms use the stationary wavelet transform (SWT). The wavelet coefficients obtained from the artefact detection algorithm are thresholded by applying a new adaptive thresholding procedure that we propose to remove artefacts in EEG signals. Its advantages over state-of-the-art artefact removal algorithms are: 

1. it can be fully automated;

2. it uses an adaptive mechanism to reduce signal distortion;

3. it is computationally inexpensive and allows real-time processing; and

4. it does not require additional electrooculogram (EOG) or electromyogram (EMG) channels, long data segments or a large number of EEG channels.

We compare the performance of different algorithms using real EEG signals and semi-simulated EEG signals (i.e., real EEG signals mixed with simulated artefacts). With semi-simulated EEG signals, we show that the proposed algorithm achieves lower signal distortion in both time and frequency domains. Next, using real EEG signals, we fully investigate and compare the performance of the hybrid BCI system in the following situations: 1) when artefacts are ignored (i.e., the original data are used); 2) when EEG segments with artefacts are rejected (i.e., the output of the system is blocked in the presence of artefacts and the system becomes unavailable); and 3) when automatic artefact removal algorithms such as the proposed algorithm and Blind Source Separation (BSS) algorithms are employed. We show that for dwell time of 0.0s (i.e., the user can activate the system any time right after he/she gazes at a letter/word), the true positive rate (TPR) achieved using the proposed artefact removal algorithm is 44.7% with 2 false positives generated per minute. This TPR value is 33.6% and 20.1% higher than those achieved when artefacts are rejected and ignored respectively. We also show that our proposed method outperforms BSS by at least 16.2%.

In the following subsections, we briefly review our self-paced hybrid BCI system, current artefact handling methods in the literature as well as the state-of-the-art of artefact removal algorithms.

### The structure of the self-paced hybrid BCI system

A hybrid BCI is defined as a system that combines a BCI with another system (such as another BCI or an eye-tracker) [13]. In this section, the overall structure of the hybrid self-paced BCI system proposed in our earlier work is presented [7]. This system combines a BCI and an eye-tracker to operate a virtual keyboard.

**Figure 1:**
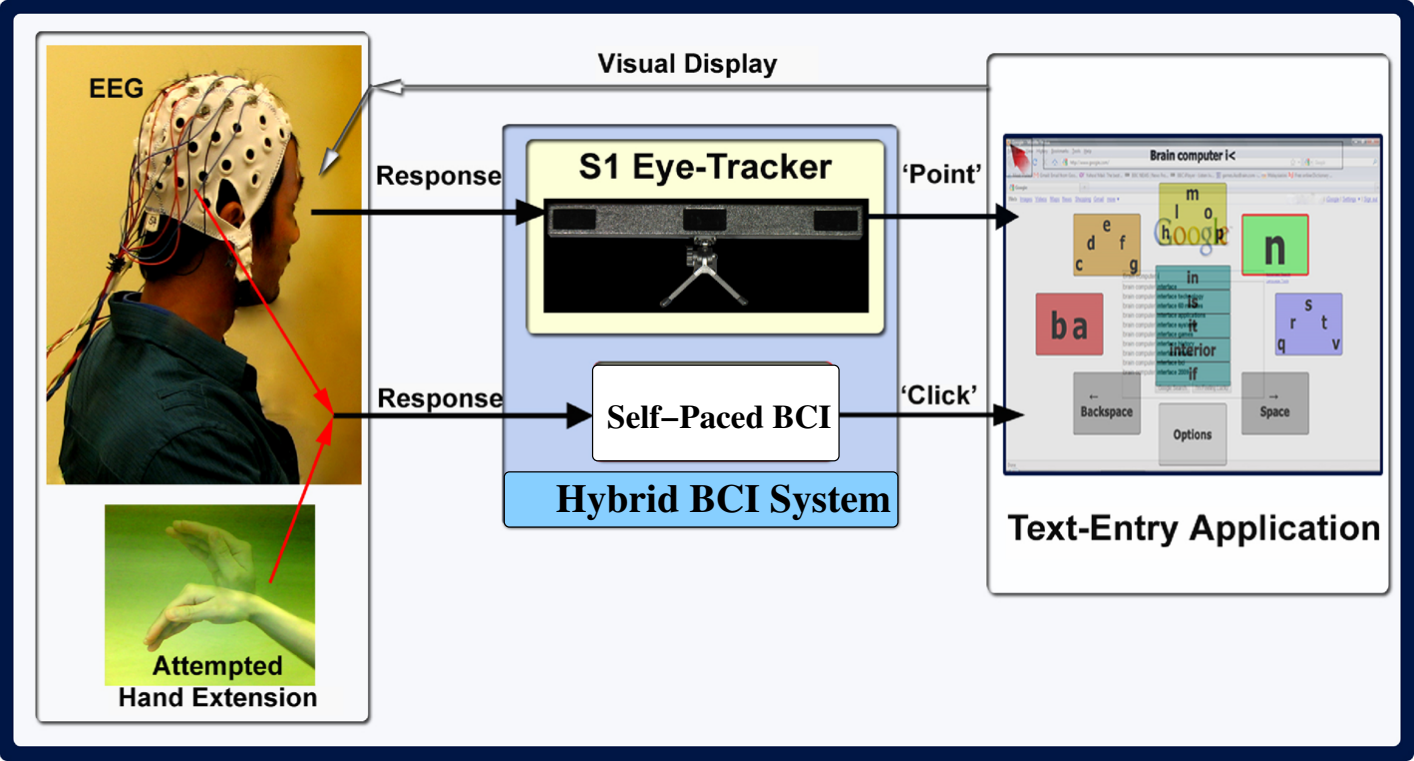
**Our Hybrid BCI System.** The hybrid BCI system proposed in [7].

Figure 1 shows the block diagram of this hybrid system. It serves as an interface between a user and a text-entry application based on a virtual keyboard called the Dynamic Keyboard [14]. The Dynamic Keyboard, which is extensively used by people with disabilities, is designed to have large selection boxes, and a word prediction functionality. The eye-tracker acts as the *pointing* device and the user’s eye gaze controls the cursor movement. The use of eye gaze is natural and fast because people often look at the object of interest before controlling it [8]. The BCI, on the other hand, acts as the *clicking* device. Its inputs are the continuous EEG signals recorded from the user’s scalp and the output is a binary control signal (i.e., it is either ‘0’ or ‘1’).

To make a selection (i.e., a *click* operation), a user has to gaze at the target for at least a specific period of time (called the *dwell time*) and then activate the self-paced BCI with a mental task (which is an attempted hand extension), as demonstrated in Figure 1. When changes in the EEG signals due to an attempted hand extension movement are detected by the signal processing unit in the BCI, a click command (an intentional control or IC) is initiated. Note that an attempted hand extension results in an imagined movement by users with movement disabilities who cannot move their hands. For able-bodied individuals, it leads to an actual hand movement [15]. Evidence from the literature shows that the patterns arising from attempted movements are very similar to those of real movements [16,17]. This evidence allows the use of real movements in our study. However, note that the attempted hand movement can be replaced by any other mental task.

Our previous study showed that increasing the dwell time (_*Tdwell*_) reduces the number of false positives [7]. This is because our design restricts the BCI’s operation to the periods during which the user’s point of gaze is within a region on the monitor that can be clicked on *and* the user gazes at that region for at least _*Tdwell*_seconds. For the rest of the time, the BCI is put in the so-called ‘*sleep*’ mode, i.e., it does not process the input EEG signals nor generates any output. By using this arrangement, we can greatly reduce the number of false positives during the NC periods, as demonstrated in [7].

The above system has one main drawback. When the users are looking at different locations of the virtual keyboard to make a selection, the amount of eye movement activity is significant. Therefore, EEG signals are more frequently contaminated with ocular artefacts compared to pure (non-hybrid) BCI systems. Hence, it is important to design an algorithm that can efficiently handle artefacts in this hybrid system.

### Artefact handling methods

A review of methods for handling EOG and EMG artefacts in BCIs shows that more than half of the 250 BCI papers studied did not report as to whether or not they had considered or handled EOG and/or EMG artefacts [9]. For those who did, three methods were generally employed: 

1. Ignore: ignoring the presence of artefacts;

2. Reject: automatic rejection of artefact-contaminated EEG segments; and

3. Remove: automatic removal of artefacts.

In a real-time self-paced BCI system, using *Ignore* or *Remove* implies that both clean and contaminated EEG signals are classified and therefore the system is available for control at all times. On the other hand, employing *Reject* indicates that the BCI system becomes unavailable for control when artefacts are present.

Rejecting contaminated EEG segments (*Reject*) is common in BCI literature. However, this approach has two major disadvantages: 

1. In the training phase, it can significantly reduce the amount of available data for training the classifier;

2. In the testing phase, it forces the BCI system into a non-responsive state for a significant portion of the time. This subsequently reduces the information transfer rate of the system.

Due to these shortcomings, *Reject* needs to be replaced by methods that do not discard any data during artefact-contaminated periods.

Unless the signal processing algorithms employed to process EEG signals are robust to the presence of artefacts, ignoring the artefacts in EEG signals (*Ignore*) is usually not an efficient approach either. This is due to the fact that artefacts affect the different frequency bands in EEG signals and therefore impact the performance of a self-paced BCI system. For example, a study conducted by Bashashati *et al.*[10] shows that the performance of the proposed self-paced BCI system deteriorates, when the data with ocular artefacts are included in the analysis. Based on the results obtained from eight participants, the amount of decrease in the true positive rate (TPR) value varied from 2.3% to 15.1% (with an average of 6.8%), when the time-normalized false positive rate (TNFPR) was set to 9 FPs/min. In another study, Fatourechi *et al.*[11] combined the use of features extracted from three neurological phenomena: movement-related potentials (MRPs), and the power of mu and beta rhythms to design a self-paced BCI system that is robust in the presence of artefacts. Using a five-fold nested cross validation, the average TPR and TNFPR achieved were 56.2% and 0.5 FPs/min for non-contaminated data and 51.8% and 2 FPs/min for artefact-contaminated data. The deterioration in some individuals was much greater, e.g., a drop of 13.2% and an increase of 0.5 FPs/min in the TPR and TNFPR, respectively, were observed in one person. The results of the above studies show that current state-of-the-art pattern recognition algorithms employed in self-paced BCI systems cannot efficiently handle artefacts. As a result, other solutions need to be explored.

A better alternative solution to handle artefacts in a self-paced BCI system is to apply automatic artefact removal algorithms to EEG segments contaminated with artefacts (*Remove*). Although removing artefacts is not straightforward and increases the complexity of the BCI system, the major advantage is that the BCI system becomes available for user’s control *at all times* including those with artefacts happen. Besides, the performance of the system may be improved if the artefact removal algorithm removes the artefacts effectively without distorting the EEG signals. In the rest of this section, we provide a brief review on artefact removal algorithms (for a more detailed review, please see [9]).

Regression analysis is widely used to remove ocular artefacts from EEG signals [18-21]. It assumes that the observed EEG signals are a linear superposition of EEG and EOG components [18]. The proportion of any EOG component that is present in the EEG signal is estimated and then removed using the least squares criterion. This method has the disadvantage of requiring the recording of source signals from the EOG channels to remove ocular artefacts. For the case of muscle artefacts, it is not straightforward to identify the source signals as these sources can originate from different muscle groups [21]. For this reason, different reference channels from multiple muscle groups are required. This in turn can greatly increase the complexity of the algorithm.

Another popular approach for artefact removal is blind source separation (BSS) [22-25], including Independent Component Analysis (ICA) algorithms [20,26-28]. These algorithms estimate the underlying sources from EEG signals recorded from electrodes. The sources related to artefacts are removed to obtain denoised EEG signals. As an example, Hung *et al.* automated the identification of EEG activities of interest using several manually identified movement-related spatial maps and used the cleaned signals in the classification of motor imagery EEG signals [26]. Halder *et al.* proposed the use of the AMUSE (Algorithm for Multiple Unknown Source Extraction) and ICA Infomax algorithms to isolate artefacts from 3-second EEG segments. A combination of support vector machines was used to classify the isolated artefacts extracted using the proposed BSS and ICA algorithms [22]. While BSS/ICA algorithms are widely used in the literature for removing artefacts, a study conducted by Wallstrom *et al.*[20] showed that these algorithms may overestimate the spectrum of artefacts and thus cause spectral distortion in EEG signals. Moreover, such methods require multi-channel data and long data epochs to produce reliable results [29].

An alternative artefact removal method is based on wavelet denoising. Stationary wavelet transform (SWT) [30] has been proposed to remove ocular artefacts (i.e., artefacts caused by eye-blinks and eye movements) from EEG signals [31-34]. In this approach, the wavelet coefficients that correspond to the lower frequency bands are thresholded to remove ocular artefacts in EEG signals. These algorithms, however, are specific to ocular artefacts and to the best of our knowledge their performance is not provided quantitatively. Besides, using the threshold selection procedure based on Stein’s unbiased risk estimate (SURE) in [33] results in over-estimation of artefacts and therefore EEG signals are over-corrected (this will be demonstrated later in this paper).

In this study, we have explored the use of SWT in removing various types of artefacts in EEG signals. The main reason is that it is computationally inexpensive and no additional EOG/EMG channels and long data segments are required. To overcome the problem encountered when using the SURE threshold selection procedure, we have proposed a new adaptive thresholding mechanism.

In the next section, we first describe the experimental procedure and the type of EEG data used in this study. Next, the artefact detection algorithm and our proposed artefact removal algorithm are discussed. Finally, the metrics used to evaluate the performance of the artefact removal algorithm is presented.

## Methods

### Experimental procedure

#### Data description

**Figure 2:**
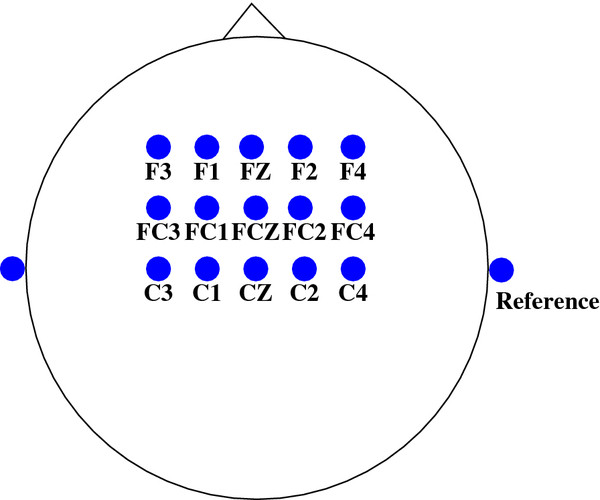
**Electrode Montage.** The EEG channels used in our system.

The experiments [7] were approved by the UBC Behavioral Research Ethics Board. We recruited seven able-bodied individuals, who did not wear glasses for this study. Their age ranges from 26 to 31. Participants gave an informed consent before participating in the experiment. Each individual was seated comfortably approximately 75 cm in front of a computer monitor and wore a 64-channel electrode cap. EEG signals were recorded from 15 electrodes placed over the motor cortex area of the brain as shown in Figure 2. Electrooculogram (EOG) signals were recorded by two pairs of electrodes placed around both eyes. Facial muscle activities were recorded by four pairs of electromyogram (EMG) surface electrodes placed symmetrically on two related facial muscles from each side of the face: zygomaticus major and corrugator supercilii. All electrodes were referenced to the linked right and left earlobes. All signals were amplified and sampled at 128 Hz using a L64 Sagura EEG amplifier system [35].

For eye-tracking, we used a Mirametrix S1 system [36]. This eye-tracker employed a single high-resolution camera to estimate the point of gaze. The eye-gaze information such as the x and y coordinates of the fixation point, the pupils’ center x and y coordinates amongst other information were recorded during the experiments.

#### Experimental protocol

Each experiment for each participant lasted for approximately 2.5 hours. At the beginning of each experiment, the eye-tracker was calibrated. Next, the participants were given approximately ten minutes to practise a text entry task with the eye-tracker and the Dynamic Keyboard so that they became more comfortable with using the system. The participants were then requested to rest for two minutes. The data recorded during this resting period were later used to determine the thresholds for the artefact detection algorithm [12].

Next, the participants were asked to type a sentence displayed by the graphical user interface (GUI), at their own speed. Once a user finished typing one sentence, a new sentence appeared and replaced the old one. This procedure was repeated until the end of the ten-minute session. The sentences were randomly selected from the ‘Phrase Set’ provided by MacKenzie and Soukoreff [37], which consisted of 500 phrases, with lengths varying from 16 to 43 characters. Each experiment consisted of three to five sessions.

To type a letter or word, each individual used eye-movements to move the cursor to the target button and then performed a hand extension to activate the self-paced BCI system. The target was selected after a hand extension movement was detected by the BCI. During data collection we replaced the self-paced BCI system with an electrical hand switch that mimicked the operation of a self-paced BCI system designed earlier by our group [38]. This switch generated an output of ‘1’ when the user performed an IC command, i.e., the user performed an attempted hand movement and pressed the switch [7]. The switch was programmed such that it had a TPR of approximately 70% at a TNFPR of about 9 FPs/min (TNFPR is the time-normalized false positive rate or the number of false positives generated per minute). These were the best performance achieved by one of our recent self-paced BCI systems based on an attempted hand extension movement [38]. Please note that during the experiment, the total TNFPR of the hybrid system was actually lower than the 9 FPs/min. This is because we designed the system so that false positives may only occur during the times when the user is gazing at a button that can be clicked on. During the periods when the user is *navigating* between selection areas, false positives are blocked and they do not result in any false selection. Hence, the total TNFPR would be lower.

Throughout the experiment, a participant could ask for a break whenever needed. Furthermore, whenever a participant felt that the eye-tracker was becoming more difficult to control, we recalibrated the eye-tracker.

#### Generating semi-simulated EEG signals

The EEG data collected from the experiments described above were used to evaluate the performance of the hybrid BCI system when various algorithms were used for artefact removal. As the exact percentage of artefacts in EEG signals is not clear, it is difficult to measure the effectiveness of different methods in terms of the amount of artefacts removed. For this reason, we have generated semi-simulated EEG signals so that the amount of artefacts and signals removed by various artefact removal algorithms can be quantified. The semi-simulated EEG signals were constructed by adding simulated artefacts to real EEG data acquired from the experiments. As the clean EEG signals, the artefacts and their mixing process are now known, evaluating the performance of different artefact removal algorithms becomes easier.

**Figure 3:**
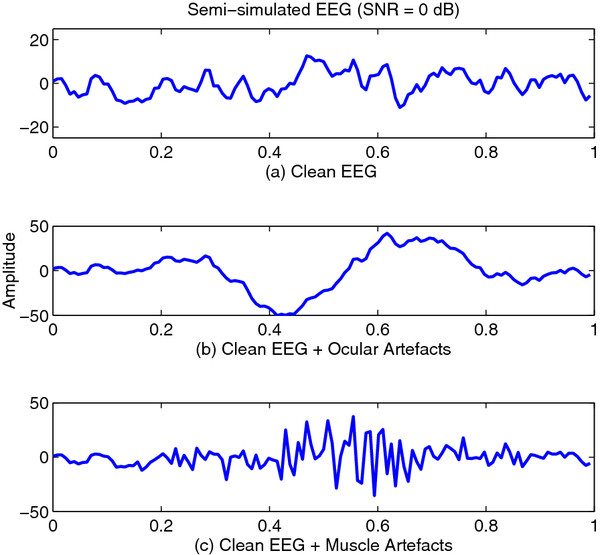
**Simulated Signals.** Examples of semi-simulated EEG signals generated from a single channel real EEG signal: **a**) clean signal; **b**) clean signal with added ocular artefacts; **c**) clean signal with added muscle artefacts.

For each of the 15 EEG channels, to generate a 1-second semi-simulated EEG signal, a 1-second clean EEG segment from each channel was mixed with artefacts. Two different types of artefacts were simulated: eye-blinks and muscle artefacts. Eye-blinks were simulated by band-pass filtering a random noise from 1 to 3 Hz. The filter was obtained using a finite impulse response (FIR) filter based on Kaiser’s window [39]. Muscle artefacts were simulated by band-pass filtering a random noise from 20 to 60 Hz using an FIR filter based on the Kaiser’s window [39]. The level of artefact contamination for each EEG channel was estimated from real EEG signals. Then, the amplitudes of the simulated artefacts were adjusted such that the semi-simulated signals have a signal-to-noise ratio (SNR) of 0 dB for the EEG channel that has the largest artefact contamination level. Figure 3 shows two examples of semi-simulated EEG signals with ocular and muscle artefacts added respectively.

To simulate real-life scenarios where EEG segments are contaminated with artefacts at different locations, each simulated artefact was shifted and mixed with each clean EEG signal to generate different semi-simulated EEG signals.

### Automatic artefact detection

**Figure 4:**
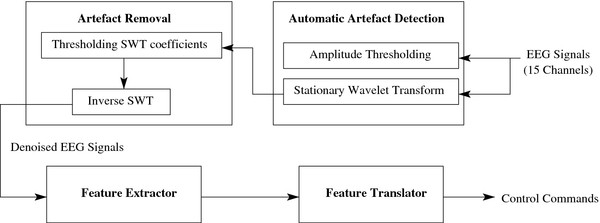
**Structure of the Proposed Self-Paced BCI System.** The structure of the proposed self-paced BCI system.

Our BCI system is composed of four main modules (see Figure 4): 

1. an artefact detection module;

2. an artefact removal module;

3. a feature extraction module; and

4. a feature classification module.

This system employs _*Ne*_=15 monopolar EEG channels. It continuously segments the EEG signals using a 1-second sliding window, with 87.5% overlap. Therefore, eight EEG segments are obtained each second. The artefact detection algorithm is first applied to each EEG segment, before that segment is processed by the artefact removal, feature extraction and feature classification modules. In the remaining part of this section, the artefact detection algorithm [12] is briefly discussed.

The automatic artefact detection algorithm is based on the stationary wavelet transform (SWT) in [12]. It only employs EEG signals acquired from the premotor and sensorimotor cortex areas of the brain. This allows us to bypass the use of additional EOG and EMG signals, as well as frontal and temporal EEG electrodes in our artefact detection module. The algorithm also has a low computational complexity because it uses a simple thresholding method for artefact detection. Furthermore, to minimize human intervention, the thresholds used in the algorithm are obtained automatically using the EEG data collected at the beginning of each experiment as the user is requested to rest and have minimal movement [12].

**Figure 5:**
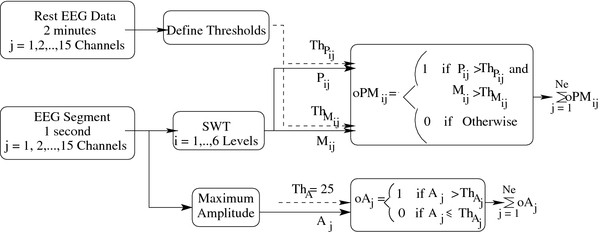
**Features Used for Automatic Artefact Detection.** Features used for automatic artefact detection [12].

The artefact detection algorithm uses the maximum amplitude of EEG signals and the SWT coefficients to detect artefacts (see Figure 5).

In Figure 5, _*Aj*_is the maximum amplitude of an EEG segment in channel *j*. In addition, _*Pij*_ and _*Mij*_ are the power and the maximum amplitude of the ^*i*th^level wavelet coefficients for the EEG channel *j* respectively as defined below:

(1) $$P_{ij}=\frac{1}{N}\overset{N}{\underset{t=1}{\sum }}a_{i,j,t}^{2}$$

(2) $$M_{ij}={\text{max}}_{t=1:N}|a_{i,j,t}|$$

where _*ai*,*j*,*t*_ is the ^*t*th^ sample of the ^*i*th^level wavelet coefficients obtained for the EEG channel *j* and *N* is the number of coefficients available.

As shown in Figure 5_*Pij*__*Mi*_, and _*Aj*_ for each EEG segment in channel *j* are computed and each of these features is compared with one of the three thresholds ( $Th_{P_{ij}}$$Th_{M_{ij}}$ and $Th_{A_{j}}$). The thresholds for these features are determined using the reference EEG signals collected when the participants were requested to rest (please see [12] for more details).

As different wavelet coefficient levels correspond to different frequency bands, the algorithm could be used to identify two major types of artefacts: (a) low frequency artefacts (e.g., ocular, electrode movement and head movement artefacts), and (b) higher frequency artefacts (e.g., facial muscle and electrode movement artefacts). The low frequency artefacts are declared present if: 

· the features of the last level of the detail coefficients and the approximation coefficients in at least NChEEG channels exceed their thresholds; or

· any of the EEG channels has a value Ajthat exceeds 25 μV

Also, the high frequency artefacts are declared to be present if the higher frequency features (_*Pij*_, _*Mij*_ for *i*=1, 2, and 3) in at least _*NCh*_EEG channels exceed the values of their corresponding thresholds.

Here, _*NCh*_ denotes the number of EEG channels that are observed to have _*Pij*_and _*Mij*_ values exceeding their corresponding thresholds. This parameter affects the sensitivity (the percentage of correctly detected segments with artefacts) and the specificity (the percentage of correctly identified artefact-free segments). The choice of _*NCh*_=0 is too stringent. Although it results in a high sensitivity value, the specificity value is often too low. In our study, we have experimentally found that _*NCh*_=5 (i.e., one third of the electrodes) provides a reasonable specificity and sensitivity values. It is clear that there is a trade-off between the sensitivity and the specificity values. For our application, a high sensitivity value (i.e., a high artefact detection rate) is more desirable because artefacts can affect the performance of the system. Those EEG segments that are falsely declared as contaminated with artefacts would not be rejected or discarded and therefore no data loss would result.

In this paper, we have integrated this artefact detection algorithm with our proposed artefact removal algorithms to denoise EEG signals. If artefacts in an EEG segment are declared as present by the artefact detection algorithm, the artefact removal algorithm is then applied to remove them, as explained in the next section.

### Artefact removal algorithm

We propose to remove the artefacts using the stationary wavelet transform (SWT) with an adaptive thresholding mechanism. As shown in Figure 4, the wavelet coefficients generated by the artefact detection module are used in our artefact removal algorithm to denoise the EEG signals. The denoised signals are obtained by performing an inverse SWT on the thresholded wavelet coefficients. The performance of the proposed algorithm is compared with those of other artefact removal algorithms such as blind sources separation (BSS) algorithms. The details of these algorithms and the performance evaluation criteria used are provided in the following subsections.

#### Background

The discrete wavelet transform (DWT) is not translation invariant. Small shifts in a signal can cause large changes in the wavelet coefficients of the signal and large variations in the distribution of energy in the different wavelet scales [30]. Besides, due to the lack of the translation invariance property, denoising with DWT sometimes introduces artefacts (small ripples) in the signal near discontinuities that are created by thresholding the wavelet coefficients [40]. A solution to the translation invariance problems is the use of a translation invariant estimation such as SWT [30].

SWT is translation invariant because there is no downsampling of data involved in the algorithm that decomposes a signal [30]. Instead, the wavelet filters are dilated at each decomposition level of the transform [30]. To remove the noise from a signal using SWT, three steps need to be performed [40]: 

1. Transform the signal into the wavelet domain;

2. Apply a thresholding function to the resulting wavelet coefficients; and

3. Transform the modified wavelet coefficients back to the original domain to obtain the denoised signal.

Therefore, when applying SWT for artefact removal, two important issues need to be taken into consideration: 1) the thresholding function used to attenuate the wavelet coefficients; and 2) the estimation procedure for obtaining the optimal threshold. These issues are discussed next.

#### Thresholding function

The thresholding function is used to remove or reduce a selected number of wavelet coefficients so as to remove artefacts from a signal. Depending on the application and the assumptions made, the large wavelet coefficients are related to either the signal of interest or to the artefacts. In our application, we assume that the artefacts that obscure the EEG signals introduce large wavelet coefficients in the wavelet domain. Hence, the wavelet coefficients (that are larger than a particular threshold *T*) correspond to noisy samples and the wavelet coefficients smaller than *T* correspond to the signal of interest. Of course, the amount of the attenuation of these coefficients depends on the thresholding function employed.

The two most widely used thresholding functions are the hard thresholding (Eq. 3) and the soft thresholding functions (Eq. 4) [40]. The hard thresholding function has a discontinuity. This discontinuity results in a bigger variance in the estimated signal (i.e., the output estimate is sensitive to small changes in the input data) [41]. The soft thresholding function on the other hand results in a bigger bias (and hence larger errors) in the estimated signal [41]. To overcome the drawbacks of both the hard and the soft thresholding, the non-negative garrote shrinkage function (Eq. 5) was proposed in [41]. This function is continuous, less sensitive to small changes in the data and has a smaller bias.

(3) $$\delta_{Hard}(x)=\left\{\begin{array}{ll} 0 & |x|\leq T\\ x & |x|>T \end{array}\right)$$

(4) $$\delta_{Soft}(x)=\left\{\begin{array}{ll} 0 & |x|\leq T\\ x-T & x>T\\ x+T & x<T \end{array}\right)$$

(5) $$\delta_{+Garrote}(x)=\left\{\begin{array}{ll} 0 & |x|\leq T\\ x-T^{2}/x & |x|>T \end{array}\right)$$

Another shrinkage function called the Smooth Sigmoid-Based Shrinkage (SBSS) function has been proposed by Atto *et al.*[42]. This function is defined as:

(6) $$\delta_{SBSS}(x)=\left\{\begin{array}{ll} 0 & |x|\leq T\\ \frac{\text{sgn}(x)(|x|-T)}{1+e^{-\tau (|x|-\lambda )}} & |x|>T \end{array}\right)$$

where sgn(*x*)=1 if *x*≥0 and sgn(*x*)=−1 if *x*<0; *T* controls the attenuation imposed on the data with large amplitudes; *λ*is the threshold height (*λ*>*T*). Finally, *τ*is the attenuation we want to impose on data with amplitudes in the interval ]*Tλ*[ and ]−*λT*[. Please see [42] for more details about the SBSS shrinkage function. The advantages of this shrinkage function are: 

1. It is smooth and it introduces small variability among coefficients with close values. Thus, it induces less error when reconstructing the signals;

2. It can control the degree of attenuation imposed on wavelet coefficients: high attenuation on the small coefficients and weak attenuation on the large coefficients.

In this paper, we investigate the different thresholding functions. Among these functions, the non-negative garrote thresholding function and the SBSS shrinkage function have not been explored in the BCI literature to remove artefacts from EEG signals and will be investigated for the first time in our paper.

#### Threshold value selection

The thresholds selected for wavelet denoising, _*Ti*_, are important as they decide the degree of attenuation imposed on both artefacts and signals. Over-estimating the thresholds results in the under-estimation of artefacts and thus, the artefacts are not completely removed from the signal of interest. On the other hand, under-estimating the thresholds results in the over-estimation of artefacts and thus, the signal of interest is over-corrected.

Two possible approaches to estimating the thresholds include: 1) estimating the thresholds based on some reference signals [31] (denoted by SWT-REF) and 2) using the so-called universal threshold proposed by [40] (Eq.7), which is denoted by SWT-UNV.

(7) $$T_{0}^{i}=\sigma_{i}\sqrt{2\text{ln}N}$$

where $T_{0}^{i}$ is the universal threshold estimated for the ^*i*th^ decomposition level wavelet coefficients _*ai*:__*σi*_ is the estimated noise variance for _*ai*:_, and *N* is the number of data samples. For this formula, _*σi*_=MADN(_*ai*:_) where MADN is the normalized version of the median absolute deviation defined below:

(8) $$\mathrm{MADN}(x)=\frac{1}{c}\text{median}(|x-\text{median}(x)|)$$

where *c*=0.6745, as this value results in an estimate that is unbiased when the data is normally distributed [43].

Both approaches provide fixed thresholds, which are not necessarily optimal. For instance, the universal threshold tends to be bigger than necessary and over-smooths the signal [41]. For our application, this implies that this threshold value fails to effectively remove artefacts.

To adaptively find the optimal thresholds, Donoho and Johnstone proposed a threshold selection procedure based on the Stein’s unbiased risk estimate (SURE) for soft-thresholding [44]. This procedure is not valid for hard thresholding because the hard thresholding function is not continuous and therefore it does not have bounded weak derivative (in Stein’s sense) [41].

When applying SWT with soft thresholding and using the SURE procedure (denoted by SWT-SURE) to remove artefacts in EEG signals, we have observed that the estimated thresholds tend to be lower than the optimal thresholds. That means the thresholds do not only remove the artefacts, but they also remove some parts of the signals as well. The evidence to support our observation will be presented in the Results section.

To overcome the problems encountered in the existing threshold selection procedures discussed above, we propose an adaptive thresholding algorithm, which is explained next.

#### Proposed adaptive SWT Denoising Algorithm - ASWTD

SWT with hard thresholding [31] and soft thresholding [33,34] have been applied in the literature to remove noise in EEG signals. These studies, however, have only focussed on ocular artefact removal. Hence, only the wavelet coefficients that correspond to lower frequency bands (i.e., up to 16 Hz) are thresholded. To the best of our knowledge, SWT has not been used to remove other artefacts such as muscle and electrode artefacts.

Our proposed algorithm, which is denoted by Adaptive SWT-based Denoising (ASWTD), is different from the above studies in two main aspects: 

1. It uses a new adaptive thresholding procedure that minimizes the effects of artefacts, while preserving the features of the signal of interest and preventing the signal from being over-corrected.

2. To remove the various EEG artefacts in a self-paced BCI system, ASWTD thresholds the wavelet coefficients at all the decomposition levels.

We also investigate four different thresholding functions (i.e., the hard, soft, non-negative garrote and SBSS thresholding functions), when the proposed procedure is employed.

**Figure 6:**
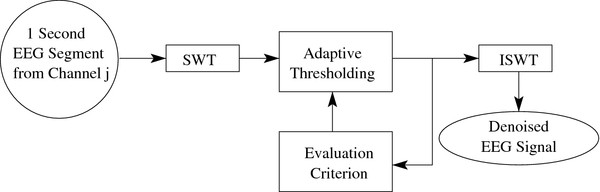
**The Proposed SWT-Based Artefact Removal Algorithm.** The basic structure of the proposed SWT-based artefact removal algorithm.

Figure 6 depicts the basic idea of the ASWTD algorithm. The thresholds are data-driven and adaptively updated. The adaptive thresholding procedure requires a performance-based criterion to decide how the thresholds should be adjusted with respect to the requirements of our application. These requirements include reducing the presence of artefacts and preserving the features of EEG signals in a computationally efficient manner.

In the proposed procedure, the evaluation criterion used to optimize the thresholds is ˚P_*ij*_, the power of the wavelet coefficients related to denoised EEG signals (see Eq. 1). ˚P_*ij*_ provides the frequency information of the signal, as the different wavelet decomposition levels correspond to the different frequency bands. If ˚P_*ij*_>*Th*_*P*_*ij*__(the same threshold value used in the artefact detection module), this means that the artefacts are still present in the signal. The threshold values of the thresholding function for each decomposition level *i* and EEG channel *j* are then modified as follows:

(9) $$T_{ij}=T_{ij}-\mu T_{ij}$$

where *μ* is the learning rate of the adaptive algorithm (0<*μ*<1). The larger the *μ*value, the faster the algorithm is in finding the optimal threshold. However, if *μ* is too large, it might result in over-estimating the artefact components and subsequently the signal distortion. We use the two values 0.1 and 0.5 for *μ*in this study. The value that results in a higher performance in the algorithm (i.e., a larger true positive rate and a larger time-normalized false positive rate in validation EEG data and less distortion in the semi-simulated EEG data) is selected. For the hard thresholding, the non-negative garrote and the SBSS functions, 0.1 is used. For the soft thresholding function, 0.5 is used.

As shown in Figure 4, ASWTD is integrated with the artefact detection module. In the artefact detection module, each of the 1-second EEG segments collected from 15 EEG channels is decomposed into five levels using SWT. As SWT is only translation invariant under circular convolution [30], any discontinuities at the borders can create large wavelet coefficients at those locations. To reduce this boundary effect, each 1-second EEG segment is extended symmetrically on the right before the *à trous algorithm* is applied. As most of the artefacts that contaminate the EEG signals are ocular artefacts, the wavelet function employed is Coiflet 3 because it resembles the shape of eye-blink artefacts [31]. Whenever artefacts are detected by the artefact detection module, ASWTD is applied to the wavelet coefficients _*aij*:_ to remove them.

A summary of ASWTD is as follows: 

1. Define the initial level-dependent threshold for each wavelet decomposition level using the universal threshold specified in Eq. 7.

2. Threshold the wavelet coefficients. The modified wavelet coefficients ${ā}_{ij:}$ correspond to artefacts. The wavelet coefficients that correspond to the EEG signals åij:are obtained by finding the difference between aij:and ${ā}_{ij:}$(i.e., ${å}_{ij:}=a_{ij:}-{ā}_{ij:}$).

3. Find the power of åij:(P̈ij) as defined in Eq. 1 and compare it to the threshold. $Th_{P_{ij}}$ While P̈ij>ThPij, the threshold value is modified according to Eq. 9.

4. Apply the inverse SWT to the final coefficient values åij:to reconstruct the denoised EEG signals.

#### Performance evaluation

It is difficult to evaluate the performance of artefact removal algorithms because a good estimate of the clean EEG activity is usually unavailable. For this reason, some studies do not quantify the performance of their proposed artefact removal algorithms. Instead, they use qualitative visual comparison, i.e., contaminated EEG signals and the corrected or denoised EEG signals are plotted and qualitatively compared [23,24,31,45]. Unfortunately, such qualitative measures are subjective. Some researchers therefore have attempted to quantify the performance by using criteria such as the ratio between the spectral density functions of the corrected and the raw EEG signals [46] and expert scoring [18].

Another approach to evaluate the performance of an artefact removal algorithm uses *simulated* EEG data. In this case, artefacts are manually added to clean EEG signals and the artefact removal algorithm is then applied to the simulated signals. With this approach, ‘clean’ EEG signals should be known. Therefore, evaluation criteria such as a correlation coefficient [25], and errors in time [20,23,25,47] or frequency domains [20] can be used to evaluate the performance. Based on this rationale, we generated semi-simulated EEG signals and investigated the performance of the different artefact removing algorithms. The performance metrics used include the signal distortion: 

1. in the time domain by using the mean square error (MSE); and

2. in the frequency domain by using the spectral distortion PSDd defined as:

(10) $$\text{PS}D_{d}=\frac{\overset{40}{\underset{f=1}{\sum }}\text{PS}D_{est}{\left(f\right)}^{2}}{\overset{40}{\underset{f=1}{\sum }}\mathrm{PS}D_{clean}{\left(f\right)}^{2}}$$

where PSDclean(f) and PSDest(f) are the spectral values at f Hz for the known clean EEG signal and the denoised EEG signal obtained using an artefact removal algorithm, respectively. The ideal value of PSDd is 1, i.e., PSDest=PSDclean. Values of PSDd<1 indicate that the algorithm over-corrects the semi-simulated EEG signals. On the other hand, if PSDd>1, the artefacts are not completely removed from the semi-simulated EEG signals or some distortion is possibly introduced by the algorithm.

Besides using semi-simulated EEG signals, we also evaluate the performance of the different artefact algorithms when applied to *real* EEG data. The performance of the system was evaluated using the true positive rate (TPR) and the time-normalized false positive rate (TNFPR) of the hybrid BCI system. TPR is the percentage of IC commands that are correctly detected by the system. False positive rate (FPR) is the percentage of false positives generated by the system during NC periods. However, FPR is NOT a good performance metric to summarize the detection performance over NC periods [7]. This is because different self-paced BCI systems may have different number of output decisions per second. Therefore, even though two systems may have the same FPR, the number of *FPs per unit of time* might be *substantially different* if their output rates are different. For example, consider systems A and B, where both A and B have an FPR of 1%. System A produces 8 decisions every second and therefore it is expected to generate approximately 4.8 FPs per minute. On the other hand, System B, which produces 16 decisions every second is expected to generate approximately 9.6 FPs per minute (i.e., twice the number of FPs generated by System A). As a result, it is more meaningful to compare the performance of different systems during NC periods using a *time-normalized measure of FPs* as proposed in [11], and defined as follows:

(11) $$\text{TNFPR}=\frac{\text{FPR}}{100}\times \text{output rate}\times 60\text{(FPs/min)}$$

To be consistent with our previous studies, a TP was declared as present when the BCI system was activated at least once in a window from 0.5s before to 1.0s after a hand switch activation [15]. Any EEG segment obtained outside the TP window was labeled as an NC trial. Therefore, any activation that occurred outside the TP window was considered as an FP. The BCI system generated 8 decisions every second. As a result, an FPR of 0.42% results in TNFPR = 0.0042×8×60=2 FPs/min (see Eq. 11).

### Feature extraction and classification algorithms

**Figure 7:**
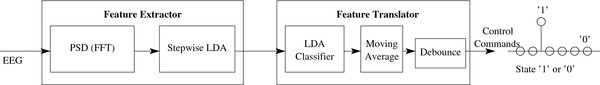
**Structure of the Feature Extraction and Classification Blocks of the BCI.** The structure of the feature extraction and classification algorithms of the self-paced BCI system [7].

After processing the EEG signals by the artefact detection and removal modules, the feature extraction and classification modules are applied next. The structure of these modules is shown in Figure 7 and their details are discussed in our previous work [7]. A brief description of their structure is as follows: First, thirty combinations of bipolar EEG signals are generated by calculating the difference between adjacent monopolar channels: Cz-C1, Cz-C2, Cz-C3, Cz-C4, C1-C2, C1-C4, C1-C3, C2-C3, C2-C4, C3-C4, FCz-Cz, FC1-C1, FC2-C2, FC3-C3, FC4-C4, Fz-FCz, F1-FC1, F2-FC2, F3-FC3, F4-FC4, FCz-FC1, FCz-FC2, FCz-FC3, FCz-FC4, FC1-FC2, FC1-FC4, FC1-FC3, FC2-FC3, FC2-FC4, FC3-FC4. Then, the power spectral density of each bipolar signal is computed by applying Fast Fourier Transform (FFT) [a window size of one second was used]. The frequency components from 1 to 35 Hz are used because they correspond to the movement related potentials as well as the mu and beta rhythms. This results in a total of 35 × 30 = 1050 features for each windowed EEG segment.

Next, the stepwise Linear Discriminant Analysis (stepwise LDA) [48] selects the features that best discriminate between the IC and NC classes. In this study, the number of features selected by stepwise LDA is subject-specific and varies from 80 to 140. Finally, Linear Discriminant Analysis (LDA) [48] is applied as a classifier [7,49]. For every participant, the EEG data collected from all sessions he/she completed (_*ns*_sessions) are divided into three parts: 

1. training data: the EEG data obtained from session 1 to ns−1, except for the last minute of the session ns−1;

2. cross-validation data: the last minute of the EEG data obtained from session ns−1;

3. testing data: all the EEG data obtained from the last ( $n_{s}^{\mathrm{th}}$) session.

The stepwise LDA and LDA classifier are trained using the training data. The value for the parameter *μ* in our proposed artefact removal algorithm is chosen using the cross-validation data. For testing the LDA classifier, all EEG segments of the last session were tested continuously in an online-like manner (i.e. as is done in an online experiment).

During testing, the LDA classifies EEG features every 0.125 seconds as a state ‘0’ (NC) or a state ‘1’ (IC). As shown in Figure 7, a moving average filter (with the length of 2 samples) and a debounce block are also employed to further improve the detection performance [11,49,50]. Debouncing the BCI output is similar to the debouncing of a physical switch. After an activation is detected by the LDA (i.e., a change from a state ‘0’ to a state ‘1’), the LDA output is set to a state ‘1’ for one sample. The next _*Tdb*_ samples, however, are forced to be the NC state ‘0’, where _*Tdb*_is the debounce period in samples. Similar to our previous study [7], a debounce component with a _*Tdb*_ of 8 decision samples is used here as well.

## Results

The performance of our proposed ASWTD is compared to those of SWT-REF, SWT-UNV, SWT-SURE, and three different blind source separation (BSS) algorithms (implemented from ICALAB toolbox [51]): 

1. SOBI (Second Order Blind Identification) [24, 25],

2. ERICA (Equivariant Robust ICA - based on Cumulants) [52] and

3. AMUSE (Algorithm for Multiple Unknown Source Extraction) [22, 25].

To be consistent with the way the EEG signals were segmented in our hybrid BCI system, the EEG signals were continuously segmented using a one-second moving window (*N*=128 samples), with an 87.5% overlap, before any BSS algorithm is applied. The mean values were removed from the 15-channel EEG segments and then the data were pre-whitened with a prewhitening matrix [53] to remove any correlations in the data. The BSS algorithms are then applied to the prewhitened EEG segments to estimate the source components of the EEG signals. The detected artefact components were removed and the denoised EEG signals were reconstructed.

We identified the artefact components automatically, based on the statistical and spectral characteristics of the source components (*s*) [39]. If one of the conditions stated below was satisfied, then artefacts were declared as present in the component: 

1. Amplitude thresholding: artefacts were declared as present, if |st|>Ths, where stis the amplitude of the tthsample of s. The threshold Thswas defined using the robust version of the ‘three sigma rule’ [43]: Ths=median(so) + 3MADN(so), where soare the amplitudes of the estimated source components of the clean reference EEG signals collected when the participants were resting.

2. Kurtosis thresholding: artefacts were declared as present if |k|>Thk, where k is the kurtosis of a source component and Thkis the threshold. Before the kurtosis of each component was computed, all one-second source components were normalized to the zero mean and a unitary standard deviation [52]. The threshold Thkwas defined as: Thk=median(ko) + 2MADN(ko), where kois the kurtosis of the normalized source components of the clean reference EEG signals. The ‘three sigma rule’ was not used in this case because we found that this particular threshold failed to detect some artefact components. Therefore, a smaller threshold value was used.

3. Spectral ratio thresholding: when high frequency artefacts were detected in the EEG signals, the artefact components were identified using a thresholding method based on the relative power spectral values, Pratio, as defined in Eq. 12. This parameter quantifies the ratio of the spectral values of the high frequency components (21 - 40 Hz) to the spectral values of the low frequency components (5 - 10 Hz).

(12) $$P_{ratio}=\frac{\overset{40}{\underset{i=21}{\sum }}P_{i}}{\overset{10}{\underset{i=5}{\sum }}P_{i}}$$

where Pi is the power spectral of a source component at the frequency i (Hz). Artefacts were declared as present in a source component, if Pratio>Thpr. The value of Thprwas determined using the robust version of the ‘three sigma rule’: Thpr=median(Po) + 3MADN(Po), where Po is the Pratioof the estimated sources of the clean reference EEG data.

To compare the performance of different artefact removal algorithms, we use different criteria depending on whether the data are semi-simulated or real EEG signals, as summarized below: 

1. Semi-simulated EEG: MSE and Spectral Distortion;

2. Real EEG: Qualitative Evaluation;

3. Real EEG: TPR and TNFPR of the Hybrid BCI System;

4. Real EEG: Inter-Trial Variability and Processing Time.

The results are now presented.

### MSE/Spectral Distortion/Qualitative Evaluation

**Figure 8:**
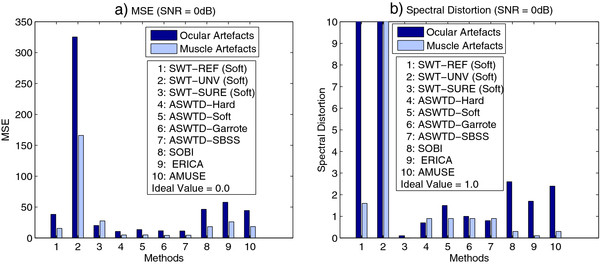
**The MSE and Spectral Distortion Obtained from Different Artefact Removal Algorithms.** The (**a**) MSE and (**b**) spectral distortion obtained from the different artefact removal algorithms when semi-simulated EEG signals are used.

**Figure 9:**
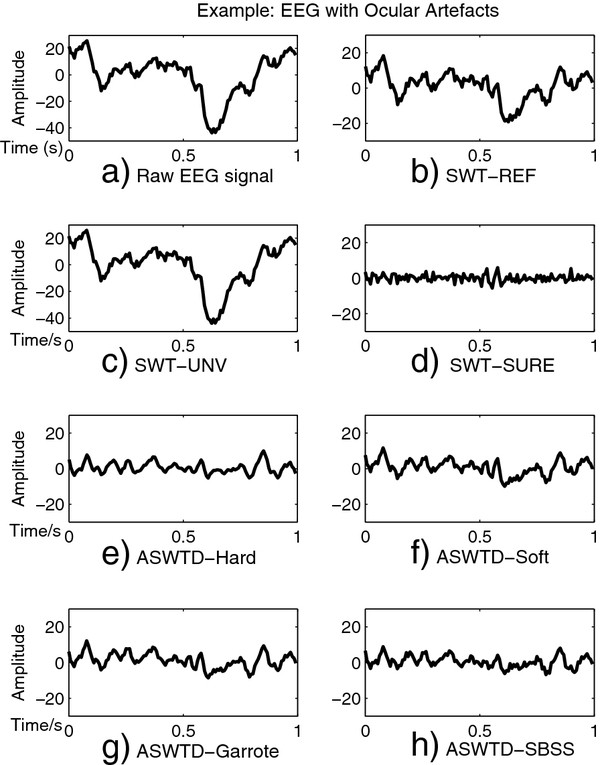
**The MSE and Spectral Distortion Obtained from Different Artefact Removal Algorithms.** The (**a**) MSE and (**b**) spectral distortion obtained from the different artefact removal algorithms when semi-simulated EEG signals are used.

Figure 8 presents the MSE and spectral distortion (*PS*_*Dd*_) for different artefact removal algorithms when semi-simulated EEG signals (with ocular and muscle artefacts added) are used. As seen in the figure, both SWT-REF and SWT-UNV have large MSE and *PS*_*Dd*_ values. In particular, the *PS*_*Dd*_values are much larger than the ideal value of 1. Figure 9 shows an example when SWT-REF and SWT-UNV are applied to a real EEG signal contaminated with ocular artefacts. The artefacts are not effectively removed when the two approaches mentioned above are used (Figure 9(b) and Figure 9(c)). The reason is that the estimated threshold values are bigger than the optimal thresholds and hence, the wavelet coefficients corresponding to the artefacts are not completely removed.

We also observe from Figure 8 that SWT-SURE has very small *PS*_*Dd*_ values (*PS*_*Dd*_<<1). For EEG signals contaminated with ocular artefacts, only the wavelet coefficients that correspond to the lower frequency bands (i.e., up to 16 Hz) are thresholded [33]. For EEG signals contaminated with muscle artefacts, the wavelet coefficients from all decomposition levels are thresholded as the artefacts affect the EEG signals in all frequency bands. Hence, a greater over-correction (a smaller *PS*_*Dd*_ value) is observed in the case of muscle artefacts. Figure 9(d) shows the denoised EEG signal obtained using SWT-SURE, when applied to the real EEG signal mentioned above. We note that the amplitude of the denoised signal is relatively small due to the over-correction.

As shown in Figure 8, the proposed ASWTD achieves smaller distortion: 1) the MSE values are smaller than other artefact removal algorithms and closer to the ideal value of 0, and 2) the spectral distortion values *PS*_*Dd*_ are close to the ideal value of 1. Among all the thresholding functions, the non-negative garrote function has the best performance. The BSS algorithms, on the other hand, have larger MSE values compared to our ASWTD. The *PS*_*Dd*_ values for the case of ocular artefacts are larger than 1, as the artefacts are not completely removed and some signal distortion may have been introduced by the algorithms. For the case of muscle artefacts, the BSS algorithms are not as efficient in isolating artefacts from the EEG signals, as compared to the case of ocular artefacts. Thus, more source components are identified as contaminated with muscle artefacts and these components are unfortunately removed [22]. This may have resulted in an over-estimation of artefacts (and larger distortion in the estimated signals). Hence, *PS*_*Dd*_ values of less than one are observed.

Figure 9 (e) - (h) presents the denoised signals obtained when ASWTD (with various thresholding functions) are used to remove artefacts in the real EEG signal Figure 9(a). Based on visual inspection, the artefacts are effectively removed by ASWTD. For the SBSS function, less information from the small coefficients is removed from the EEG signals and more information from the large coefficients (corresponding to artefacts) has been removed. Hence, the denoised signal obtained shows slightly more details (and therefore is less smooth) compared to the rest.

**Figure 10:**
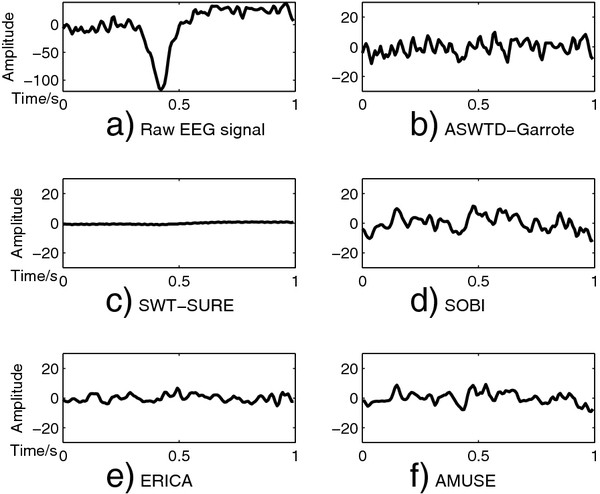
**Denoised EEG Signals Obtained Different Artefact Removal Algorithms (Ocular Artefacts).** The real EEG signal contaminated with ocular artefacts is shown in **a**). The denoised EEG signals obtained using the different artefact removal algorithms are shown in **b**) - **f**): **b**) ASWTD-Garrote; **c**) SWT-SURE; **d**) SOBI; **e**) ERICA; and **f**) AMUSE.

**Figure 11:**
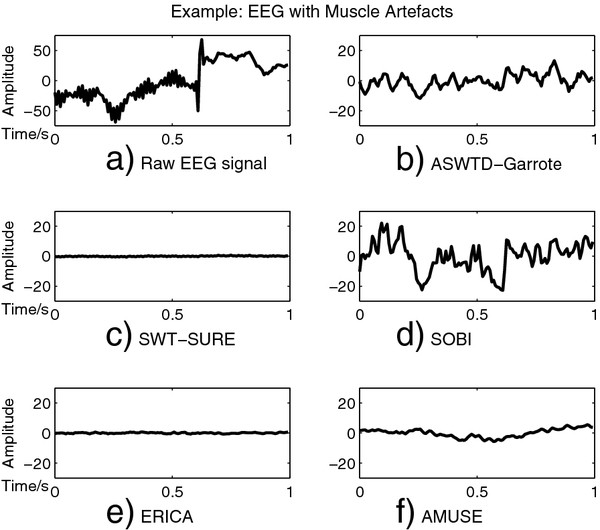
**Denoised EEG Signals Obtained Different Artefact Removal Algorithms (Muscle Artefacts).** The real EEG signal contaminated with muscle artefacts is shown in **a**). The denoised EEG signals obtained using the different artefact removal algorithms are shown in **b**) - **f**): **b**) ASWTD-Garrote; **c**) SWT-SURE; **d**) SOBI; **e**) ERICA; and **f**) AMUSE.

Examples of applying SWT-SURE, ASWTD and BSS algorithms to real EEG signals are shown in Figure 10 and Figure 11. The raw EEG segments are contaminated with an eye-blink and fEMG artefacts respectively. As shown in Figure 10, SOBI, AMUSE and ERICA remove the artefacts to a certain extent. In Figure 11(d), however, SOBI fails to completely remove the artefacts. For AMUSE, ERICA and SWT-SURE, the EEG signals are over-corrected and the distortion is observed in the denoised signals. On the other hand, ASWTD with the non-negative garrote thresholding function gives the best results. It has smaller signal distortion as well as a smaller variance between the two estimated denoised signals.

### TPR/TNFPR of the hybrid BCI

Table 1 compares the average performance achieved by the hybrid BCI system for seven individuals, when different artefact handling methods and dwell times (_*Tdwell*_) are used (note that a dwell time of 0.0s implies that the user can select a target immediately once he gazes at it). All the real EEG segments obtained from the last session are included in the analysis (including those contaminated with artefacts). We consider the following three artefact handling methods (explained in Section 2): 

1. Ignore: No artefact handling is employed;

2. Reject: Contaminated EEG segments are rejected;

3. Remove: An artefact removal algorithm (ASWTD, SWT-SURE, SOBI, ERICA or AMUSE) that denoises contaminated EEG segments is applied.

For ASWTD, different thresholding functions are used: 

1. ASWTD_Hard: ASWTD + hard thresholding

2. ASWTD_Soft: ASWTD + soft thresholding

3. ASWTD_Garrote: ASWTD + non-negative garrote thresholding

4. ASWTD_SBSS: ASWTD + SBSS thresholding

A two-way Analysis of Variance (ANOVA) [54] was carried out to examine the statistical significance of the results. ANOVA showed that the mean performances of the hybrid BCI system with different artefact handling methods and different dwell times were significantly different at a significance level of 0.01.

As shown in Table 1, the hybrid BCI system with *Ignore* has an average TPR = 11.1% and TNFPR = 2.0 FPs/min, when the dwell time is 0.0s. As the dwell time increases to 0.5s, and finally to 1.0s, the TPR increases to 34.3% and then to 62.8% (for the same TNFPR).

When *Reject* is used, many EEG segments are rejected and blocked by the system due to the presence of artefacts. The explanation is as follows. The EEG data recorded from seven participants during the last session contained an average of 88 ± 19 IC trials and 2595 ± 698 NC trials (IC trials = the number of attempted hand extension executed; NC trials = the number of 1-second EEG segments obtained outside the TP window, as defined earlier). Approximately 48.4 ± 38.8% of IC and 90.2 ± 11.4% of NC trials were contaminated with artefacts. Rejecting these trials means that these data are discarded and not presented as inputs to the system. Therefore, whenever artefacts are detected, the availability of the BCI for control is significantly reduced. This may lead to generating many false negatives (i.e., missed true activations) because many IC trials are blocked due to artefacts. Hence, both the TPR and TNFPR values are small and the results are not significantly different for various dwell times.

On the other hand, *Remove* allows the users to have more control over the BCI system, as the system is operational even in the presence of artefacts. Besides, this approach reduces the effects of artefacts and achieves a better performance when compared to *Ignore* and *Reject*. This performance improvement is especially significant, when the value of _*Tdwell*_is small. For example, when dwell time is 0.0s, the TPR achieved using ASWTD_Garrote is 44.7% , which is more than 20% of those of *Ignore* and *Reject*. As the dwell time increases, the performance difference between the methods decreases. The reason is that increasing the dwell time reduces the availability of the system to only those periods for which a selection might happen. Thus, the system is put in the so-called ‘inactive’ mode more frequently and the effects of artefacts on the system’s performance are significantly reduced.

ASWTD using different thresholding functions also outperforms SWT-SURE and other BSS algorithms. Among all the thresholding functions, the non-negative garrote thresholding achieves the best performance, i.e., TPR =44.7% and TNFPR =2.0 FPs/min. The TPR increases steadily to 73.1% when the dwell time increases to 1.0s.

In Table 1, the performance of ASWTD is obtained from the BCI classifier trained using *both clean and denoised EEG trials*. We also investigate the performance of ASWTD_Garrote, when the BCI classifier is trained using *only clean EEG trials* (denoted by *BC*_*Iclean*_). The results are presented in Table 2. Note that the TPR values obtained in Table 2 are lower than those in Table 1 because a smaller number of EEG trials are available to train *BC*_*Iclean*_ due to artefact contamination. When *Ignore* is used and the dwell time is 0.0s, TPR = 11.1% and TNFPR = 2.0 FPs/min are obtained. ASWTD_Garrote, on the other hand, removes artefacts in contaminated EEG trials and successfully improves the TPR values from 11.1% to 22.2% (at the same TNFPR). The contribution to the improvement comes entirely from those EEG trials with artefacts, because the proposed algorithm does not operate on clean EEG trials (i.e., the performance from of both artefact handling methods remain the same when only clean EEG trials are evaluated). The results in Table 2 suggest that when artefacts are ignored, the artefacts results in a change in the quality of the EEG signals and therefore affect the performance of *BC*_*Iclean*_. ASWTD_Garrote successfully minimizes the effects of artefacts and improves the classifier’s performance. When a larger number of trials are used in training the classifier (Table 1), ASWTD_Garrote achieves even higher TPR values (at the same TNFPR).

**Table 1:** Comparing the Performance of Different Artefact Handling Methods

**Method**	**(TPR:%, TNFPR:FPs/min)**				
	***T*_*dwell*^= 0.00^_**	***T*_*dwell*^= 0.25^_**	***T*_*dwell*^ = 0.50^_**	***T*_*dwell*^ = 0.75^_**	***T*_*dwell*^= 1.00^_**
*Ignore*	(11.1, 2.0)	(11.7, 2.0)	(24.3, 2.0)	(48.0, 2.0)	(62.8, 2.0)
*Reject*	(24.6, 1.5)	(26.3, 1.4)	(28.1, 1.4)	(30.7, 1.3)	(28.7, 1.1)
SOBI	(28.5, 2.0)	(33.9, 2.0)	(42.3, 2.0)	(54.5, 2.0)	(66.4, 2.0)
ERICA	(17.1, 2.0)	(20.1, 2.0)	(31.3, 2.0)	(45.4, 2.0)	(60.4, 2.0)
AMUSE	(27.4, 2.0)	(30.5, 2.0)	(37.0, 2.0)	(56.4, 2.0)	(72.6, 2.0)
SWT-SURE	(16.2, 2.0)	(19.7, 2.0)	(27.7, 2.0)	(38.0, 2.0)	(54.2, 2.0)
ASWTD_Hard	(36.4, 2.0)	(34.3, 2.0)	(43.1, 2.0)	(53.0, 2.0)	(69.7, 2.0)
ASWTD_Soft	(44.0, 2.0)	(46.5, 2.0)	(51.1, 2.0)	(62.3, 2.0)	(70.3, 2.0)
**ASWTD_Garrote**	**(44.7, 2.0)**	**(48.8, 2.0)**	**(51.7, 2.0)**	**(60.8, 2.0)**	**(73.1, 2.0)**
ASWTD_SBSS	(36.7, 2.0)	(37.7, 2.0)	(48.0, 2.0)	(56.4, 2.0)	(71.8, 2.0)

Comparing the performance of the hybrid BCI system with the different artefact handling methods and dwell times.

**Table 2:** Comparing the Performance of *Ignore* and ASWTD_Garrote

**Method**	**(TPR:%, TNFPR:FPs/min)**				
	***T*_*dwell*^= 0.00^_**	***T*_*dwell*^ = 0.25^_**	***T*_*dwell*^= 0.50^_**	***T*_*dwell*^ = 0.75^_**	***T*_*dwell*^= 1.00^_**
*Ignore*	(11.1, 2.0)	(11.7, 2.0)	(24.3, 2.0)	(48.0, 2.0)	(62.8, 2.0)
ASWTD_Garrote	(22.2, 2.0)	(23.7, 2.0)	(35.4, 2.0)	(52.5, 2.0)	(66.6, 2.0)

Comparing the performance of the hybrid BCI system using *Ignore* and ASWTD_Garrote when the classifier is trained using only clean EEG trials.

### Inter-Trial Variability/Processing Time

When an artefact removal algorithm shows a large trial-by-trial variability in the amplitudes of the denoised signals, this might suggest that the algorithm is not efficient in removing artefacts. Possible causes of such a large inter-trial variability could be that: 

1. the algorithm does not completely remove artefacts or

2. the algorithm sometimes removes the artefacts efficiently, but sometimes over-corrects the EEG signals or does not completely remove the artefacts.

Here, we quantify the inter-trial variability in the amplitudes of the denoised EEG signals (estimated using various artefact removal algorithms) when applied to real EEG signals by finding the standard deviation of: 

1. the variance of each estimated denoised EEG signals (σvar)

2. the difference between the maximum and minimum value of each denoised EEG signals (σmax−min)

The results are presented in Table 3. Evidently, the _*σvar*_and _*σmax*−*min*_are large when the artefacts are ignored because of the large differences between the amplitudes of clean and contaminated EEG signals. ASWTD, however, has a significantly smaller _*σvar*_ and _*σmax*−*min*_ values. The BSS algorithms have larger _*σvar*_and _*σmax*−*min*_values because the denoised EEG signals estimated by these algorithms are less consistent. For example, in Figure 10(d), SOBI successfully removes the ocular artefacts, whereas in Figure 11(d), SOBI fails to completely remove the muscle artefacts. This results in a larger inter-trial variability.

**Table 3:** Inter-Trial Variability and Processing Time

**Method**	**_**σmax−min**_**	**_**σvar**_**	**Time (ms)**
*Ignore*	39.3	950.9	0
SOBI	12.4	77.4	54
ERICA	14.2	127.6	23
AMUSE	12.5	82.0	12
ASWTD_Garrote	4.2	8.4	30

Comparing the _*σvar*_and _*σmax*−*min*_values, and the processing time obtained using the different artefact handling methods.

Besides inter-trial variability, we also examine another performance metric that needs to be taken into consideration for online implementation: the processing time required to run the artefact algorithms (see the last column of Table 3). In this study, all algorithms were run in Matlab 2009b environment. For SWT, the Rice Wavelet Toolbox from RICE University was used [55]. The processor used was an 2.93 GHz Intel (R) Core i7 870. As shown in Table 3, all algorithms require no more than 60 ms to process a 1-second EEG segment with 15 channels, indicating their suitability for online applications.

## Discussion

This paper proposes a fully automated algorithm to remove artefacts from EEG signals and subsequently improve the performance of our hybrid BCI system. Specifically: 

1. we propose an adaptive thresholding method based on SWT to remove various artefacts in EEG signals. It is shown that the proposed method (ASWTD) greatly improves the performance of the hybrid BCI system and reduces signal distortion and

2. we investigate the effects of using different thresholding functions in the performance of ASWTD.

In the following subsections, more details about the above claims are provided.

### Comparison of different artefact handling methods

We have investigated and compared the performance of our hybrid BCI system, when different artefact handling methods are used to denoise the real EEG data. The performance is evaluated using a pseudo-online testing paradigm, where all real EEG data (both clean and contaminated) are included in the testing. Such testing provides us with a better understanding of the system’s performance in a real-world online application, where artefacts are present in the EEG signals.

We need to emphasize the importance of the system having a low TNFPR. A low TNFPR ensures that the system does not cause too much frustration for users. This is because users are in an NC state for most of the time when using the system. Also, it is usually easier to deal with a missed IC command than with a false activation (i.e., an FP). For example, in a text-writing application, a false positive results in selecting the wrong letter/word. Consequently, the user has to initiate additional commands to *de-select* the wrong letter/word and then select the correct desired letter/word. On the other hand, in the case of a missed IC, the user only has to issue the IC command again. Therefore, it is important to lower the TNFPR as much as possible.

Table 1 and Table 2 show that artefacts can affect the BCI system’s performance. If artefacts are ignored (*Ignore*), the system has a low TPR value, especially when the dwell time is small. The rejection of contaminated EEG segments (*Reject*), on the other hand, reduces the amount of time for which the hybrid BCI system is available for control. In addition, this approach rejects IC trials contaminated with artefacts, which results in lower TPR values (Table 1). The drawbacks of *Ignore* and *Reject* signify the need to minimize the effects of artefacts by applying artefact removal algorithms. As shown in Table 1, *Remove* greatly improves the performance of the hybrid BCI system.

Our study demonstrated that the proposed artefact removal algorithm ASWTD can improve the hybrid BCI system’s performance in two ways: 

1. ASWTD_Garrote reduces the effects of artefacts and improves the performance of the hybrid BCI system. This is when the BCI classifier is trained with clean EEG trials only (see Table 2);

2. ASWTD_Garrote increases the number of clean EEG trials available for training the BCI classifier. Both the clean and denoised EEG trials are used to train the classifier. This further increases the detection performance of the hybrid BCI system (see Table 1).

ASWTD also has another advantage: a smaller dwell time can be used when the algorithm is incorporated into the hybrid BCI system. Thus, the user does not have to gaze at the target for too long to make a selection. For example, ASWTD_Garrote achieves a TPR of 48.8% at a TNFPR of 2 FPs/min when the dwell time is 0.25s. This performance is as good as the one achieved by *Ignore* but when the dwell time is 0.75s (TPR = 48.0%, TNFPR = 2 FPs/min).

### Comparison of different artefact removal algorithms

Our results shows that ASWTD outperforms SWT-SURE, SOBI, ERICA and AMUSE. More specifically, it achieves: 

1. lower MSE values and less spectral distortion when semi-simulated EEG signals with ocular and muscle artefacts are used (see Figure 8);

2. larger TPR values when real EEG signals are used (see Table 1); and

3. smaller inter-trial variability in the amplitudes of the denoised EEG signals when real EEG signals are used (see Table 3).

**Figure 12:**
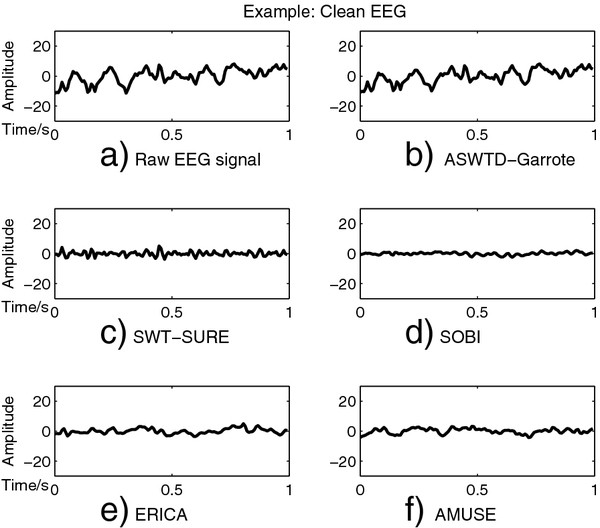
**Denoised EEG Signals Obtained Different Artefact Removal Algorithms (Without Artefacts).** The real clean EEG signal is shown in **a**). The denoised EEG signals obtained using the different artefact removal algorithms are shown in **b**) - **f**): **b**) ASWTD-Garrote; **c**) SWT-SURE; **d**) SOBI; **e**) ERICA; and **f**) AMUSE.

As the proposed artefact removal algorithm introduces less distortion in EEG signals, false artefact detection (i.e., the artefact detector falsely detects artefacts in a clean EEG signal) may not pose too much of a problem. An example is shown in Figure 12, where a clean EEG segment is processed using ASWTD_Garrote, SWT-SURE, SOBI, ERICA and AMUSE. The denoised signal obtained using ASWTD has less distortion while the other algorithms over-correct the EEG signal. Also, when semi-simulated EEG signals are used (see Figure 8), ASWTD also achieves smaller MSE and *PS*_*Dd*_ values (which are closer to the ideal values).

SWT-SURE does not perform as well because the estimated thresholds often lead to the over-estimation of artefacts and hence it removes some EEG features (*PS*_*Dd*_<<1, for semi-simulated EEG signals). The other three BSS algorithms also do not perform as well as ASWTD. A possible reason is that BSS algorithms are not usually applied to short EEG segments (i.e., 1 second). The length of data segment used in most artefact-removal studies is at least 3 seconds [22,24,26,45]. According to [45], if the amount of data used in a BSS algorithm is not sufficient, the decomposition results may not be robust. Hence, in this study, the BSS algorithms are less effective in removing artefacts and have a bigger inter-trial variability in the estimated denoised EEG signals when compared to ASWTD. The use of longer data segments can improve the effectiveness of the BSS algorithms in removing artefacts.

In terms of processing time (in the Matlab environment), all algorithms require no more than 60 ms to process a 1-second segment collected from 15 EEG channels. The proposed hybrid BCI system processes EEG segments every 125 ms (i.e., 8 outputs are generated every second). Therefore, all signal processing algorithms have to be executed within 125 ms. The artefact detection and FFT feature extraction algorithms take approximately 4 ms and 3 ms, respectively, to process a 1-second EEG segment with 15 channels. That means, when the proposed artefact detection and removal algorithm is incorporated into the BCI, the total processing time for all signal processing algorithms is less than 50 ms, which is suitable for real-time processing. We expect these numbers to be significantly improved if the algorithm is implemented in C + + environment, which is more suitable for real-time applications.

### Comparing different thresholding functions

Of the four thresholding functions investigated for our proposed ASWTD, the non-negative garrote thresholding with the proposed adaptive thresholding procedure achieves the best performance (in terms of MSE, *PS*_*Dd*_, and TPR values). This function is less sensitive to small changes in the data and has a smaller bias compared to hard and soft thresholding functions [41]. Hard thresholding does not perform as well (probably because it is discontinuous and the variance of the estimated denoised signal is larger than that achieved by other thresholding functions). Besides, hard thresholding sets the values of wavelet coefficients that are larger than their corresponding thresholds to zero. Hence, all the wavelet coefficients that correspond to artefacts are removed from the EEG signals. It might also remove from the EEG signals some features that are captured in these large coefficients. Thus, its *PS*_*Dd*_ values are slightly less than unity when applied to semi-simulated EEG signals. Other thresholding functions, on the other hand, do not completely remove those large wavelet coefficients that correspond to artefacts. For example, for non-negative garrote and soft thresholding, the wavelet coefficients that are larger than *T* are reduced by a certain amount depending on the coefficient values. This in turn preserves more features in the EEG signals.

## Conclusions

In summary, we have demonstrated that the proposed artefact removal method ASWTD_Garrote (SWT with the non-negative garrote thresholding function and an adaptive thresholding mechanism) improves the TPR values of the hybrid system (at the same TNFPR) and a smaller dwell time can be used. The proposed method outperforms other artefact handling methods and provides the following advantages: 

· it does not require long data segments or a large number of EEG channels;

· it allows real-time processing;

· it does not require additional EOG/EMG channels to detect and remove artefacts;

· it allows adaption to the characteristics of a given signal, resulting in minimal distortion in EEG signals even in the case of false artefact detection;

· it can be applied to all artefact types; and

· it is fully automated.

In our future work, we will look into methods that automatically select the optimal wavelet function for the proposed algorithm. It is also of interest to extend the proposed algorithm (which is univariate) to a multivariate version and find out if and how it can improve the effectiveness of the algorithm in denoising EEG signals. In addition, we will investigate algorithms to adaptively update the classifier of the hybrid BCI system such that the TNFPR value remains low in online experiments. Finally, we will implement the proposed hybrid BCI system online and investigate the usability and performance of the system during online studies.

## Competing interests

The authors declare no competing interest.

## Author’s contributions

XY designed the hybrid BCI system, proposed the algorithm, carried out the experiments, collected and analyzed the data. XY drafted the paper. MF assisted in the interpretation of the results and the evaluation of the performance of the system. RKW and GEB supervised the development of the study. All authors reviewed and approved the final manuscript.

## References

[B1] Donchin E Spencer KM Wijesinge R The mental prosthesis: assessing the speed of a P300-based brain computer interface IEEE Trans Rehabil Eng 2000 8 2 174 179 10.1109/86.847808 10896179

[B2] d R Millan J Renkens F Mourino J Gerstner W Brain-actuated interaction Artif Intell 2004 159 241 259 10.1016/j.artint.2004.05.008

[B3] Scherer R Müller GR Neuper C Graimann B Pfurtscheller G An asynchronously controlled EEG-based virtual keyboard: improvement of the spelling rate IEEE Trans Biomed Eng 2004 51 6 979 1307 10.1109/TBME.2004.827062 15188868

[B4] Middendorf M McMillan G Calhoun G Jones KS Brain-computer interfaces based on the steady-state visual evoked response IEEE Trans Rehabil Eng 2000 8 2 211 214 10.1109/86.847819 10896190

[B5] Pfurtscheller G Guger C Müller G Krausc G Neuper C Brain oscillations control hand orthosis in a tetraplegic Neurosci Lett 2000 292 211 214 10.1016/S0304-3940(00)01471-3 11018314

[B6] Mason SG Birch GE A brain-controlled switch for asynchronous control applications IEEE Trans Biomed Eng 2000 47 10 1297 1307 10.1109/10.871402 11059164

[B7] Yong X Fatourechi M Ward RK Birch GE The design of a point-and-click system by integrating a self-paced brain-computer interface with an eye-tracker IEEE JETCAS Special Issue on Brain Machine Interface 2011 1 4 590 602

[B8] Jacob RJK The use of eye movements in human-computer interaction techniques: what you look at is what you get ACM Trans Inf Syst (TOIS) 1991 9 2 152 169 10.1145/123078.128728

[B9] Fatourechi M Bashashati A Ward RK Birch GE EMG and EOG artifacts in brain computer interface systems: a survey Clin Neurophysiol 2006 118 3 480 494 17169606 10.1016/j.clinph.2006.10.019

[B10] Bashashati A Nouredin B Ward R Lawrence P Birch G Effect of eye-blinks on a self-paced brain interface design Clin Neurophysiol 2007 118 1639 1647 10.1016/j.clinph.2007.03.020 17466588

[B11] Fatourechi M Ward RK Birch GE Performance of a self-paced brain-computer interface on data contaminated with eye-movement artifacts and on data recorded in a subsequent session Comput Intelligence Neurosci 2008 2008 13 10.1155/2008/749204 PMC2386957 18497872

[B12] Yong X Fatourechi M Ward RK Birch GE Automatic artefact detection in a self-paced brain-computer interface system IEEE PACRIM 2011 IEEE, Victoria, Canada 403 408

[B13] Pfurtscheller G Allison BZ Brunner C Bauernfeind G Solis-Escalante T Scherer R Zander TO Muller-Putz G Neuper C Birbaumer N The hybrid BCI Front Neurosci 2010 2 3 1 11 10.3389/fnpro.2010.00003 PMC2891647 20582271

[B14] Dynamic Keyboard|CanAssist http://www.canassist.ca/dynamic-keyboard

[B15] Bashashati A Fatourechi M Ward RK Birch GE User Customization of the Feature Generator of an Asynchronous Brain Interface Ann Biomed Eng 2006 34 6 1051 1060 10.1007/s10439-006-9097-5 16783660

[B16] Birch GE Bozorgzadeh Z Mason SG Initial online evaluations of the LF-ASD brain-computer interface with able-bodied and spinal-cord subjects using imagined voluntary motor potentials IEEE Tran Neural Syst Rehabil Eng 2002 10 4 219 224 10.1109/TNSRE.2002.806839 12611359

[B17] Beisteiner R Hollinger P Lindinger G Lang W Berthoz A Mental representations of movements. Brain potentials associated with imagination of hand movements Electroencephalography Clin Neurophysiology 1995 96 2 183 193 10.1016/0168-5597(94)00226-5 7535223

[B18] Schlögl A Keinrath C Zimmermann D Scherer R Leeb R Pfurtscheller G A fully automated correction method of EOG artifacts in EEG recordings Clin Neurophysiol 2007 118 98 104 10.1016/j.clinph.2006.09.003 17088100

[B19] Moretti DV Babiloni F Carducci F Cincotti F Remondini E Rossini PM Salinari S Babiloni C Computerized processing of EEG-EOG-EMG artifacts for multicentric studies in EEG oscillations and event-related potentials Int J Psychophysiology 2003 47 199 216 10.1016/S0167-8760(02)00153-8 12663065

[B20] Wallstrom GL Kass RE Miller A Cohn JF Fox NA Automatic correction of ocular artifacts in the EEG: a comparison of regression-based and component-based methods Int J Psychophysiology 2004 53 105 119 10.1016/j.ijpsycho.2004.03.007 15210288

[B21] Gasser T Schuller JC Gasser US Correction of muscle artefats in the EEG power spectrum Clin Neurophysiol 2005 116 2044 2050 10.1016/j.clinph.2005.06.002 16043401

[B22] Halder S Bensch M Mellinger J Bogdan M Kubler A Birbaumer N Rosenstiel W Online artifact removal for brain-computer interfaces using support vector machines and blind source separation Comput Int Neurosci 2007 2007 10 10.1155/2007/82069 PMC2234090 18288259

[B23] Ting KH Fung PCW Chang CQ Chan FHY Automatic correction of artifact from single-trial event-related potentials by blind source separation using second order statistics only Med Eng Phys 2006 28 780 794 10.1016/j.medengphy.2005.11.006 16406675

[B24] Joyce CA Gorodnitsky IF Kutas M Automatic removal of eye movement and blink artifacts from EEG data using blind component separation Psychophysiology 2004 41 2 313 325 10.1111/j.1469-8986.2003.00141.x 15032997

[B25] Crespo-Garcia M Atienza M Cantero J Muscle artifact removal from human sleep EEG by using independent component analysis Ann Biomed Eng 2008 36 3 467 475 10.1007/s10439-008-9442-y 18228142

[B26] Hung CI Lee PL Wu YT Chen LF Yeh TC Recognition of motor imagery electroencephalography using independent component analysis and machine classifiers Ann Biomed Eng 2005 33 8 1053 1070 10.1007/s10439-005-5772-1 16133914

[B27] Jung TP Humphries C Lee TW Makeig S McKeown MJ Iragui V Sejnowski TJ Extended ICA removes artifacts from electroencephalographic recordings Adv Neural Inf Process Syst 1998 10 894 900

[B28] Iriarte J Urrestarazu E Valencia M Alegre M Malanda A Veteri C Artieda J Independent component analysis as a tool to eliminate artifacts in EEG: a quantitative study J Clin Neurophysiology 2003 20 4 249 257 10.1097/00004691-200307000-00004 14530738

[B29] Delorme A Makeig S EEGLAB: an open source toolbox for analysis of single-trial EEG dynamics including independent component analysis J Neurosci Meth 2004 134 9 21 10.1016/j.jneumeth.2003.10.009 15102499

[B30] Mallat S A Wavelet Tour of Signal Processing 1998 Academic Press, USA

[B31] Zikov T Bibian S Dumont GA Huzmezan M Ries CR A wavelet based de-noising technique for ocular artifact correction of the electroencephalogram EMBS IEEE, Houston, USA 2002 2002

[B32] Ramanan SV Kalpakam NV Sahambi JS A novel wavelet based technique for detection and de-noising of ocular artifact in normal and epileptic electroencephalogram ICCCAS IEEE, Houston, USA 2004 2004

[B33] Krisnaveni V Jayaraman S Anitha L Ramadoss K Removal of ocular artifacts from EEG using adaptive thresholding of wavelet coefficients J Neural Eng 2006 3 338 346 10.1088/1741-2560/3/4/011 17124338

[B34] Kumar PS Arumuganathan R Sivakumar K Vimal C Removal of Ocular Artifacts in the EEG through Wavelet Transform without using an EOG Reference Channel Int J Open Problems Compt Math 2008 1 3 13

[B35] L64 EEG/PSG Data Acquisition Amplifier System, Dr. Sagura Medizintechnik http://l64.sagura.royalmedicalsystems.com/

[B36] Mirametrix Research, S1 Eye-tracker, 2010 http://www.mirametrix.com/s1-eye-tracker.html

[B37] MacKenzie IS Soukoreff RW Phrase sets for evaluating text entry techniques Ext. Abstracts on Human Factors in Computing Systems CHI 2003 2003 ACM Press, New York, USA 754 755

[B38] Bashashati A Towards development of a 3-State Self-Paced Brain Computer Interface System PhD in electrical and computer engineering 2007 University of British Columbia 10.1155/2007/84386 PMC2234253 18288260

[B39] Delorme A Sejnowski T Makeig S Enhanced detection of artifacts in EEG data using higher-order statistics and independent component analysis NeuroImage 2007 34 1443 1449 10.1016/j.neuroimage.2006.11.004 17188898 PMC2895624

[B40] Coifman RR Donoho DL Translation-Invariant De-Noising NeuroImage 1995 Springer-Verlag, Berlin, Germany 125 150

[B41] Gao HY Wavelet shrinkage denoising using the non-negative garrote J Comput Graphical Stat 1998 7 4 469 488

[B42] Atto AM Pastor D Mercier G Wavelet shrinkage: unification of basic thresholding functions and thresholds Signal, Image and Video Process 2009 5 11 28

[B43] Maronna RA Martin RD Yohai VJ Robust Statistics: Theory and Methods 2006 Wiley, England

[B44] Donoho DL Johnstone IM Adapting to unknown smoothness via wavelet shrinkage J Am Stat Assoc 1995 90 432 1200 1224 10.1080/01621459.1995.10476626

[B45] Jung TP Makieg S Humphries C Lee EW Mcjeown MJ Iragui V Sejnowski T Removing electroencephalographic artifacts by blind source separation Psychophysiology 2000 37 163 178 10.1016/S0167-8760(00)00088-X 10731767

[B46] Schöloegl A Ziehe A Müller KR Automated ocular artifact removal: comparing regression and component-based methods Available Nat Precedings 2009 2009 24

[B47] Quiroga RQ Garcia H Single-trial event-related potentials with wavelet denoising Clin Neurophysiol 2003 114 376 390 10.1016/S1388-2457(02)00365-6 12559247

[B48] Lachenbruch PA Discriminent Analysis 1975 Hafner Press, New York

[B49] Bashashati A Ward RK Birch GE Towards development of a 3-State Self-Paced Brain-Computer Interface Comput Intelligence Neurosci 2007 2007 8 10.1155/2007/84386 PMC2234253 18288260

[B50] Borisoff JF Mason SG Bashanti A Birch GE Brain-computer interface design for asynchronous control applications: improvements to the LF-ASD asynchronous brain switch IEEE Trans Biomed Eng 2004 51 6 985 992 10.1109/TBME.2004.827078 15188869

[B51] ICALAB for Signal Processing http://www.bsp.brain.riken.go.jp/ICALAB/ICALABSignalProc

[B52] Barbati G Porcaro C Zappasodi F Rossini PM Tecchio F Optimization of an independent component analysis approach for artifact identification and removal in magnetoencephalographic signals Clin Neurophysiol 2004 115 1220 1232 10.1016/j.clinph.2003.12.015 15066548

[B53] Cichocki A Amari SI Adaptive Blind Signal and Image Processing: Learning Algorithms and Applications 2002 West Sussex, England

[B54] Ledolter J Hogg RV Applied Statistics for Engineers and Physical Scientists 2009 Pearson Prentice Hall, NJ

[B55] Rice Wavelet Toolbox | Rice DSP http://dsp.rice.edu/software/rice-wavelet-toolbox

